# Ammonia Exposure Induced Cilia Dysfunction of Nasal Mucosa in the Piglets

**DOI:** 10.1155/2020/1705387

**Published:** 2020-05-25

**Authors:** Qiankun Wang, Mengyao Wang, Chun Liu, Longhui Huang, Yun Gao, Mei Yu, Shuhong Zhao, Xiaoping Li

**Affiliations:** ^1^Key Lab of Agricultural Animal Genetics, Breeding and Reproduction of Ministry of Education, College of Animal Science and Technology, Huazhong Agricultural University, Wuhan 430070, China; ^2^College of Engineering, The Cooperative Innovation Center for Sustainable Pig Production, Huazhong Agricultural University, Wuhan 430070, China

## Abstract

As one of the main environmental stressors commonly found in closed pig houses, ammonia poses high risks to the well-being of humans and animals. This study is aimed at assessing the toxicity of ammonia exposure (80 ppm for 12 days) on the nasal mucosa in piglets. Firstly, we found that after ammonia exposure, the number of white blood cells significantly increased and the serum levels of cytokine IL-4 were significantly decreased. Then, histological analyses showed significant thickening of nasal mucosa and excessive mucus production in the exposure group. Finally, RNA-seq analyses demonstrated that the ammonia exposure disturbed the transcriptome of nasal mucosa which revealed 176 upregulated genes and 426 downregulated genes. GO and KEGG pathway enrichment analysis of the DEGs showed that the upregulated genes were mainly related to neutrophil chemotaxis and immune response, while 80 out of the 426 downregulated genes including CCDCs, CFAPs, DNAHs, and TEKTs were enriched in the microtubule cytoskeleton and cilium morphogenesis/movement. All these results indicated that ammonia exposure induces nasal mucosal hyperplasia and cilia dysfunction, as well as a systemic inflammatory response in piglets. These findings provide new evidence for understanding the damage mechanism of ammonia on the nasal mucosa.

## 1. Introduction

Intensive farming of livestock is usually confronted with the problem of poor air quality. Ammonia is usually found in high concentrations, which seriously affects the indoor air quality in closed pig houses, particularly in winter when the ventilation is largely inadequate [1]. It has been reported that high concentration of ammonia has negative impacts on the growth, physiological functions, and immunity of animals [2–5]. For example, pigs showed decreases in weight gain and food consumption when exposed to 100 ppm ammonia for 4–5 weeks [6]. Increased numbers of neutrophils in the nasal lavage fluid were observed in pigs exposed to lower concentrations (25 or 50 ppm) of ammonia for 6 days [7]. Similarly, when broilers were exposed to high concentration of ammonia for several weeks, they also showed decreased feed intake and daily weight gain, because exposure to ammonia may trigger oxidative stress and autophagy and interfere with the nutrient absorption and immune function of the small intestinal mucosa and result in cardiac damage of broilers [8, 9].

Ammonia dissolves in moisture on tissues or mucous membranes to form ammonium hydroxide and, thus, is an irritant of the upper respiratory tract, nose, and eyes, negatively affecting the health of animals and humans [10]. When inhaled into the airway, ammonia primarily affects the mucous membranes of the nose and is retained in the mucus of the nasal mucosa as NH_4_^+^. Representative examples of nasal histopathology from mice have shown that continuous exposure to ammonia can cause severe suppurative rhinitis with marked epithelial degeneration, necrosis, and sloughing, as well as obvious mucosal and submucosal suppurative inflammatory infiltrate [11].

The nasal mucosa surface is covered by a pseudostratified mucosal epithelium, which is composed of multiple cell types including basal, ciliated, and secretory cells (goblet and serous cells) [12]. These epithelial cells form a highly regulated physical and immune barrier for defense against exogenous stimuli [13]. Healthy nasal mucosa surface is covered by two layers of liquid produced by the epithelial goblet cells and submucosal glands. The surface mucous layer can entrap inhaled particles and pathogens, and the second watery layer is around the cilia and facilitates ciliary beating and efficient mucus clearance [14]. Under normal conditions, the nasal mucosa epithelium has a strong regeneration ability, but long-term or repeated challenge by exogenous stimuli may alter the normal structure and function of nasal mucosa. For example, in human or animal models with asthma, cystic fibrosis (CF), idiopathic bronchiectasis, or chronic obstructive pulmonary disease (COPD), the nasal mucosa epithelium is usually disrupted, accompanied by markedly upregulated mucus production and secretion [15, 16]. Thus, it can be inferred that the local microenvironment and normal function of the airway play an important role in defending against the damage caused by ammonia exposure.

Although the effects of ammonia exposure on airway pathology have been investigated for over decades, its influence on gene expression is not well understood. The present study is aimed at further elucidating the impacts of ammonia exposure on the transcriptome of the nasal mucosa. Weaned piglets were exposed to 80 ppm ammonia for 12 days in air-pollutant exposure chambers, and the haematological analyses implied a systemic inflammatory response of the piglets after exposure. Then, by using hematoxylin and eosin (H&E) and alcian blue- (AB-) periodic acid Schiff (AB-PAS) staining, we studied the effect of ammonia on the structure of the nasal mucosa. Transcriptome analysis by RNA-seq was carried out to further explore the molecular mechanism of ammonia toxicity. A large number of genes related to inflammatory response were identified to be upregulated after ammonia exposure, but those genes related to the function of cilium were downregulated. Our results are expected to provide valuable clues for understanding the effects of ammonia exposure on the airway of pigs.

## 2. Methods and Materials

### 2.1. Ammonia Exposure Experiment and Sample Collection

In total, six 7-week-old healthy castrated hybrid (Yorkshire×Landrace) male piglets (14 kg in weight) were randomly divided into two groups (*n* = 3 per group). Then, the two groups were exposed in air-pollutant exposure chambers with ammonia concentrations of 80 ppm (D12 group) and <1 ppm (NC group) for 12 days (8 h for each day). All animals were allowed to adapt to the new environment for 3 days before any interventions were carried out, and during the whole experiment, the animals were supplied with free food and water. Compressed liquid ammonia was put into the 30 m^3^ exposure chamber with a specific flow speed through a valve. At the same time, appropriate ventilation was supplied to maintain the desired indoor ammonia concentrations. Two air agitators were used to diffuse the ammonia evenly indoor, and three ammonia gas detectors (DTN220B-NH_3_, Shanghai Weitai Technology) were equipped in the exposure chamber to dynamically monitor the ammonia concentration. In order to reduce the interference of ammonia produced by the feces of the piglets, the chambers were immediately cleaned after the exposure experiment every day.

The piglets were executed after 12 h of fasting on the 12th day, and two precaval vein blood samples from each piglet were collected. 3 mL of blood sample with anticoagulant was used for a routine blood test (three classifications), which was stored on ice and sent to be analyzed within 2 h. Another 5 mL of blood sample without anticoagulant was used for cytokine detection by Enzyme-Linked Immunosorbent Assay (ELISA), which was immediately (within 1 h) sent to the laboratory and centrifuged at 5000 rpm for 15 min, and then the serum samples were collected and stored at −20°C for ELISA assays. The samples collected from the nasal septum were used for histopathological analysis by H&E and AB-PAS and immunohistochemical staining. Meanwhile, the samples from the nasal mucosa were also collected and stored in liquid nitrogen immediately for RNA extraction to perform RNA-seq and qPCR amplification.

### 2.2. Analysis of Haematological Parameters

Haematological parameters are good indicators of the physiological status of farm animals. In order to assess the impact of ammonia exposure on the health of piglets, the total white cell count and the amounts of lymphocytes, monocytes, and granulocytes in the blood were analyzed with the routine blood test (three classifications) at the end of exposure experiments, and each sample was measured twice. In addition, the anti-inflammatory cytokines such as interleukin-4 (IL-4), interleukin-10 (IL-10), and interferon-*γ* (IFN-*γ*) in the serum were measured for their protein levels by commercially available ELISA kits (IL-4: CSE0002, IL-10: CSE0003, and IFN-*γ*: CSE0004; Beijing 4A Biotech) twice for each sample according to the manufacturer's instructions. Data from the D12 group were compared with those of the NC group using the Student *t*-test. We considered *p* < 0.05 as statistically significant.

### 2.3. Histological Analysis of the Nasal Mucosa

In order to check the morphological changes in the nasal mucosa after ammonia exposure, H&E staining was firstly performed on the nasal septum. Samples were immersed in 4% phosphate-buffered paraformaldehyde firstly, embedded in paraffin, sectioned (4 *μ*m thick), and stained with hematoxylin and eosin. In order to assess the levels of intracellular mucous glycoconjugates in the nasal mucosa under ammonia exposure, the sections were stained with AB-PAS after deparaffinization with xylene and rehydrated in an alcohol gradient, and the detailed process referred to a previously described protocol [17].

Previous reports have indicated that MUC5AC and MUC5B are the predominant mucins expressed in goblet cells and submucosal glands, respectively [18]. We therefore checked their expression levels in the nasal mucosa by immunohistochemical analysis. The nose sections were fixed by 4% formaldehyde and then stained with anti-MUC5AC antibody (1 : 100, BA3293; Boster Corporation) and anti-MUC5B antibody (1 : 100, D261005; Sangon Biotech (Shanghai)) using the SABC IHC kits (SA1022/SA1028/SA1020, Boster Corporation (Wuhan)). Following immunohistochemical staining, the sections were counterstained with hematoxylin and mounted. Pictures of the sections were captured using an Olympus BX53 microscopy imaging system. Then, thickness of the H&E staining was quantified by Image-Pro Plus 6.0 software. For each group, three to five stained sections from each individual were analyzed. Finally, real-time PCR was performed to further verify the results.

### 2.4. RNA Isolation, Library Construction, and RNA Sequencing

Total RNA was isolated from the nasal mucosa of six individuals using a TRIzol reagent (Invitrogen, San Diego, CA, USA), and then the quality and the concentration were checked. A total of 3 *μ*g RNA for each sample (6 samples in total) was sent to the Novogene Company (Beijing, China) for library construction and sequencing. The library quality was assessed on the Agilent Bioanalyzer 2100 system, and then the library was sequenced on an Illumina HiseqTM PE125 platform to generate 125 bp paired-end reads.

### 2.5. Identification of Differentially Expressed Genes (DEGs)

Raw data (raw reads) of the FASTQ format were first processed through in-house Perl scripts to obtain clean data (clean reads) by removing the reads containing an adapter, poly-N > 10%, and other low-quality reads. At the same time, Q20, Q30, and GC content of the clean data was calculated. All the subsequent analyses were based on the clean data. Pig scrofa11.1 genome draft (reference genome) and gene model annotation files were downloaded from Ensemble (http://asia.ensembl.org/index.html), and then the index of the reference genome was built using Bowtie v2.2.5, and paired-end clean reads were aligned to the reference genome using TopHat v2.0.14. The BAM files from TopHat were analyzed by RSeQC software to get the initial alignment information. HTSeq v0.6.0 was used to count the reads mapped to each gene, and the gene expression levels in the transcriptomes were quantified using Cufflinks software. The abundance of the expressed genes was calculated as expected fragments per kilobase of transcript per million fragments mapped (FPKM) [19]. Pearson correlation coefficient was calculated by the R package before differentially expressed gene (DEG) analysis in order to exclude the abnormal samples. Differential expression analysis of the two groups was performed using the DESeq R package (1.20.0). The resulting *p* values were adjusted using the Benjamini-Hochberg approach for controlling the false discovery rate. Genes with adjusted *p* values (*q* value) < 0.05 as detected by DESeq [20] were assigned as DEGs. Quantitative real-time PCR (qPCR) was conducted to confirm the gene expression at the transcription level for nine selected DEGs in normal and ammonia-exposed nasal mucosa samples. Span-intron primers of the nine genes were designed for qPCR (primers provided in Supplementary Table S1). PCR reaction conditions were denatured at 94°C for 2 min, followed by 39 cycles at 94°C for 30 s, 60°C for 30 s, and 72°C for 30 s. Standard curves were constructed using serially diluted cDNA to ensure the amplification efficiency, and melting curves were generated to confirm the specificity of amplification [21]. The expression level of a target gene was normalized to the mRNA level of GAPDH, and quantitative analysis of the data was performed using the 2^-*ΔΔ*Ct^ method [22].

### 2.6. Functional Annotation of DEGs

We used the FPKM of DEGs to draw the hierarchical clustering heat map of the NC and D12 groups in the nasal mucosa. In order to excavate the association between the gene function and phenotypes in the two groups, KEGG pathways and Gene Ontology (GO) annotations were analyzed in the DAVID database (V6.8, http://david.abcc.ncifcrf.gov/) by functional clustering and annotation tools, and the input DEGs of pigs were firstly transformed to human homologous genes through the BioMart tool in the Ensemble database. The KEGG pathways and GO terms with corrected *p* values lower than 0.05 were considered as significantly enriched. Besides, the DEGs were also subjected to gene set enrichment analysis (GSEA) by the WebGestalt [23], an online analytical software (http://www.webgestalt.org/). The uploaded text should include the |log2FC| > 1 and padj < 0.05, and the DEGs were sorted by “padj.”

## 3. Results

### 3.1. Ammonia Exposure Caused a Systemic Inflammatory Response of Piglets

**Figure 1:**
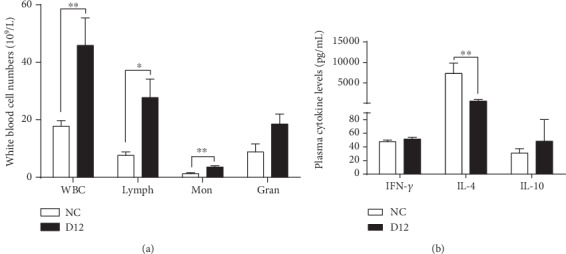
White blood cell counting results and anti-inflammatory cytokine levels in serum. (a) Total white blood cell counts. WBC: white blood cells; Lymph: lymphocytes; Mon: monocytes; Gran: granulocytes. (b) The interleukin-4 (IL-4), interleukin-10 (IL-10), and interferon-*γ* (IFN-*γ*) levels in serum were detected by ELISA. Data were presented as mean ± SD. ^∗^*p* < 0.05; ^∗∗^*p* < 0.01.

Since haematological parameters are good indicators of the physiological status of farm animals, we firstly compared these parameters in piglets of the exposure group with those of the NC group and found that the total numbers of white blood cells (*p* < 0.01), lymphocytes (*p* < 0.05), and monocytes (*p* < 0.01) were all significantly increased in the exposure group (Figure 1(a)).

We also determined the expression levels of several anti-inflammatory cytokines such as IL-4, IL-10, and IFN-*γ* in the serum by ELISA (Figure 1(b)) to further assess the health status of these piglets. The results showed that ammonia exposure did not change the protein levels of IL-10 and IFN-*γ*. However, the expression of IL-4 was nearly abolished after ammonia exposure. These results suggested that the piglets displayed a systemic inflammatory response to ammonia exposure.

### 3.2. Ammonia Exposure Induced Nasal Mucosa Hyperplasia and Mucus Overproduction

**Figure 2:**
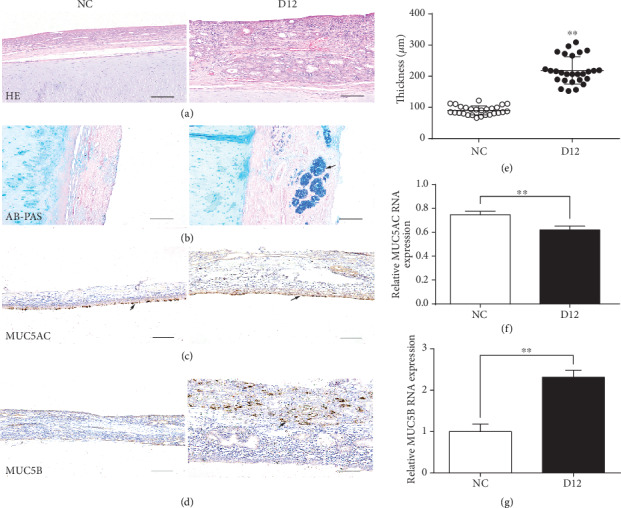
Histopathological analysis of nasal mucosa. (a) Results of H&E staining of nasal mucosa. Bars = 50 *μ*m. (b) Results of AB-PAS staining of mucus produced by goblet cells and submucosal glands (arrow). Bars = 50 *μ*m. (c) Immunohistochemical staining of MUC5AC protein in the surface epithelium of nasal mucosa (arrow). Bars = 50 *μ*m. (d) Immunohistochemical staining of MUC5B protein in nasal mucosa (arrow). Bars = 50 *μ*m. (e) Statistical analysis of thickness of nasal mucosa. The pictures used for statistics have the same size, pixel value, shooting conditions, and magnification for each group, and 10 to 15 stained sections from each group were analyzed. (f) qPCR results of MUC5AC. (g) qPCR results of MUC5B. Data from (f) and (g) were analyzed with Student's *t*-test, and the values were presented as mean ± SD. ^∗^*p* < 0.05; ^∗∗^*p* < 0.01.

Ammonia readily dissolves in water to form ammonium hydroxide. Previous studies have shown that ammonia and ammonium hydroxide are corrosive and can rapidly penetrate the eye to cause permanent injury [10]. When inhaled into the animal respiratory tract, it primarily affects the mucous membranes of the upper respiratory tract. Thus, we detected the histological changes in the nasal mucosa by H&E and AB-PAS staining (Figure 2). In the NC group, the epithelium was smooth and tightly packed, with a small number of submucosal glands, but in the exposure group, the mucosa was significantly thickened, and a part of epithelial structure was damaged (shedding), with the appearance of many vacuole-like structures and submucosal glands (Figures 2(a) and (e)). AB-PAS staining was used to further explore the changes of mucus production under ammonia exposure in the nasal mucosa (Figure 2(b)). The staining signal was relatively weak in the NC group, with only some blue-stained particles being observed along the mucosal surface epithelium, while in the D12 group, a much stronger staining signal could be observed in the submucosa (magenta and blue), indicating that ammonia exposure caused mucus overproduction due to the hyperplasia of submucosal glands. Previous reports have indicated that MUC5AC and MUC5B are the predominant mucins expressed in goblet cells and submucosal glands, respectively [24]. We therefore checked their expression levels in the nasal mucosa by immunohistochemical staining and qPCR. In the nasal mucosa, the expression of MUC5AC was observed to be repressed in the surface epithelium after ammonia exposure for 12 days (Figures 2(c) and (f)); however, MUC5B showed an opposite expression pattern, as its expression was significantly increased in the submucosa after ammonia exposure (Figures 2(d) and (g)).

### 3.3. Ammonia Exposure Disturbed the Gene Expression Profiles of Nasal Mucosa

Transcriptomes of the nasal mucosa were constructed from the individuals of the NC group and the D12 group by RNA sequencing. After paired-end sequencing, more than 100 M raw reads with the length of 125 bp were obtained for each sample. After quality control, the content of clean data from each sample was above 12 Gb with Q20 > 96%, Q30 > 92%, and GC content ≈ 53% (for details, see Table S2). When the clean reads were mapped to the pig scrofa11.1 reference genome, the overall mapping rate was about 72% in the nasal mucosa. Analysis of the FPKM distribution showed about 7% high-expression genes (FPKM > 60) and over 61% low-expression genes in the nasal mucosa (FPKM < 1).

In order to identify the genes with variations in expression after ammonia exposure, random comparisons were performed by DESeq among the NC and D12 groups. Finally, a total of 602 differentially expressed genes (DEGs; 176 upregulated and 426 downregulated; *q* value < 0.05) were identified in the D12 group in comparison with the NC group. The volcano plot of DEGs is shown in Figure S1.

### 3.4. Ammonia Exposure Induced Nasal Cilia Dysfunction

**Figure 3:**
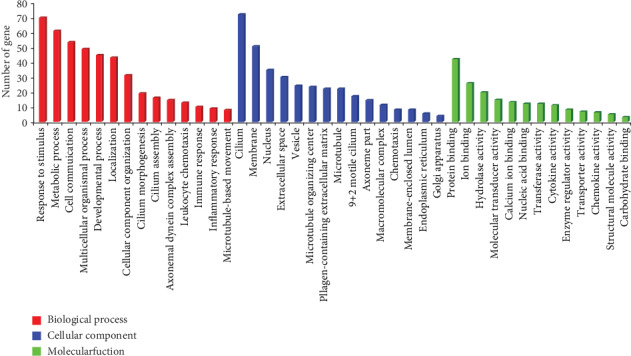
GO analysis for biological process, cellular component, and molecular function.

In summary, our study suggested that ammonia exposure changes the mucosal gene expression profiles. To investigate the functions of DEGs, the Gene Ontology (GO) enrichment analysis based on three categories (biological process, cellular component, and molecular function) was conducted by WebGestalt (Figure 3). The enriched GO terms with the largest number of DEGs from the three categories were the response to stimulus (*n* = 70), cilium (*n* = 72), and protein binding (*n* = 42).

**Figure 4:**
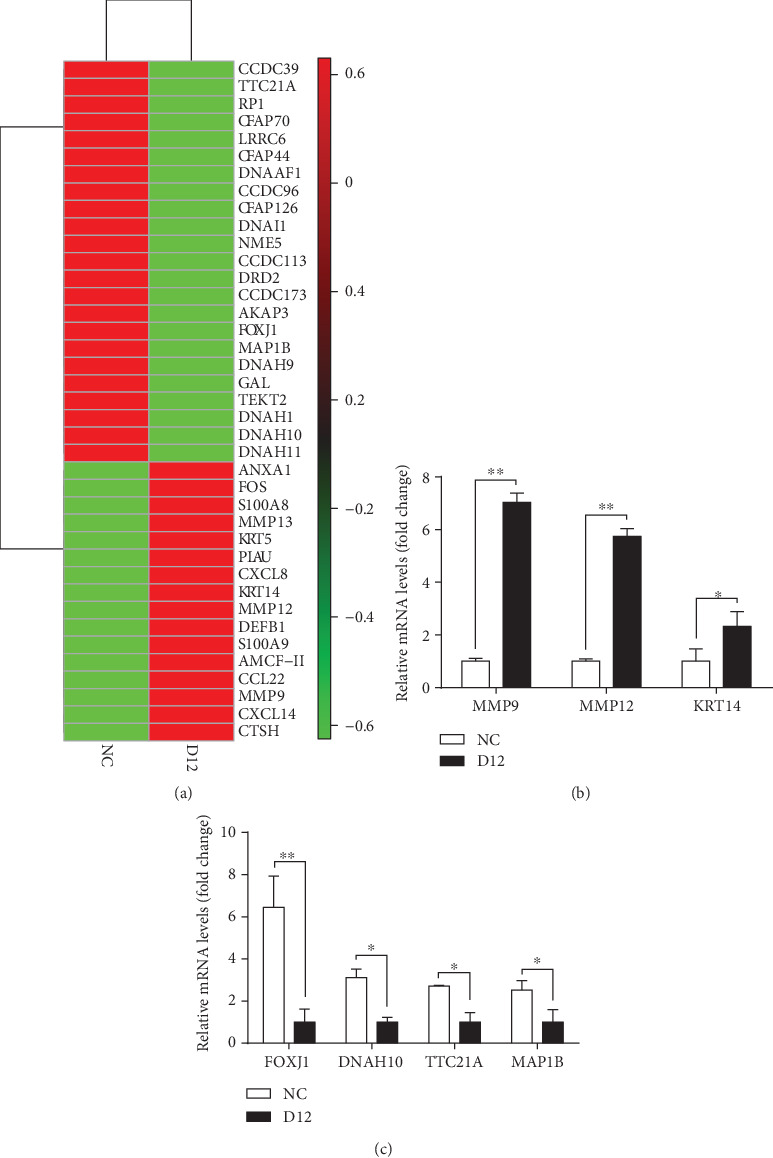
The downregulated genes related to cilium morphogenesis. (a) The heat map of DEGs related to nasal mucosal remodeling and cilium morphogenesis. Red squares indicate upregulation and green squares represent downregulation after ammonia exposure. (b) qPCR results of the upregulated genes related to nasal mucosal remodeling. (c) qPCR results of the downregulated genes related to cilium morphogenesis. All data were analyzed with Student's *t*-test, and the values are expressed as mean ± SD. ^∗^*p* < 0.05; ^∗∗^*p* < 0.01.

In order to further explore the biological events occurring in the nasal mucosa of piglets after ammonia exposure, we performed GO and KEGG pathway analysis in the DAVID database using the 176 upregulated and 426 downregulated DEGs, respectively. Surprisingly, 80 out of 426 downregulated genes including CCDCs, CFAPs, DNAHs, and TEKTs were enriched in the microtubule cytoskeleton and cilium morphogenesis/movement (Table 1). However, the upregulated genes such as cytokines (*AMCF-II*, *CCL22*, *CCL3L1*, *CXCL14*, *CXCL8*, and *IL1B1*) and matrix metalloproteinases (MMPs: *MMP9*, *MMP12*, and *MMP13*) were mainly related to neutrophil chemotaxis and immune response. It was worthwhile that these genes (*MMP9*, *MMP13*, *S100A8*, *S100A9*, *CSF3*, *FOS*, and *CXCL8*) are mainly involved in the IL-17 signaling pathway. The heat map of DEGs related to nasal mucosal remodeling and cilium morphogenesis is illustrated in Figure 4(a). Based on the RNA-seq data, seven genes related to nasal mucosal remodeling (*MMP9*, *MMP12*, and *KRT14*) and cilium morphogenesis (*FOXJ1*, *DNAH10*, *TTC21A*, and *MAP1B*) were selected to further check their expression by qPCR (Figures 4(b) and 4(c)). The results showed that they had the same expression trend as that obtained from RNA-seq data. Therefore, it could be concluded that ammonia stimuli cause cilia dysfunction in the nasal mucosa and trigger the autoimmune defense mechanism to promote the healing of the epithelium.

## 4. Discussion

Ammonia from animal production causes serious environmental pollution and adversely affects the ecosystem and health of animals and humans [10]. This study systematically explored the toxicity of 80 ppm ammonia exposure on the nasal mucosa of piglets at the histological and transcriptomic levels. Ammonia exposure for 12 days significantly disrupted the structure of the nasal mucosa and induced inflammatory responses in piglets. Damage of the mucosa structure will greatly increase the susceptibility of animals to pathogenic microbes and induce respiratory diseases, further affecting the productivity and survival of piglets.

The number of white blood cells is an important indicator of health status, which is usually increased in the systemic inflammatory response. In the present study, we found that the number of white blood cells was significantly increased by ammonia exposure for 12 days, indicating that ammonia stimulation causes severe inflammatory responses in piglets. In order to limit the potentially injurious effects of excess inflammatory responses, the host secretes immunoregulatory molecules such as anti-inflammatory cytokines. Hence, we detected the serum levels of three anti-inflammatory cytokines IFN-*γ*, IL-10, and IL-4 and found that IL-4 was nearly abolished after ammonia exposure. IL-4 promotes Th2 lymphocyte development and inhibits the LPS-induced proinflammatory cytokine synthesis and, thus, plays an important role in wound repair [25]. Previous studies have shown that IL-4^−/−^ and IL-4R*α*^−/−^ mice were highly susceptible to endometrial damage after primary *C. trachomatis* infection [26]. Therefore, decrease in IL-4 after continuous ammonia exposure may increase the susceptibility of piglets to pathogens.

Since the nasal mucosa has direct contact with the outside environment and can express multiple immune factors such as cytokines and mucins, it is considered as the first defensive line of the respiratory tract [24]. It can be concluded that injury of the mucosal epithelium results in a nasal inflammatory response. Excessive mucus secretion/production is a significant feature of inflammatory airway diseases. In this study, positive AB-PAS staining signals were mainly found in the submucosal glands (SMGs) in the exposure group when compared with the NC group, indicating that ammonia exposure induces mucus overproduction in nasal mucosa, which may impede gas flow in the respiratory tract. Studies in mice have demonstrated the important roles of MUC5AC and MUC5B in the homeostasis of airway epithelial cells and in respiratory tract innate immunity [27]. In this study, the expression of MUC5AC decreased in the surface epithelium of nasal mucosa, while that of MUC5B increased in the SMGs after ammonia exposure. Taking the results of AB-PAS staining and the MUC5AC/MUC5B expression analysis together, we speculated that ammonia exposure induces hyperplasia of submucosal glands. The decreased expression of MUC5AC further indicated the decrease in goblet cells owing to the shedding of the partial nasal epithelium. The abnormal expression of *MUC5AC* and *MUC5B* induced by ammonia exposure may result in the functional abnormality of nasal mucosa in piglets and increase the susceptibility of animals to pathogenic microbes.

In this study, we also observed the shedding of the mucosal epithelium and mucosal hyperplasia in piglets in the exposure group, indicating that ammonia exposure induces the damage of the nasal mucosa and at the same time triggers the autoimmune defense mechanism to promote the healing of the epithelium. Lineage-tracing studies have shown that both KRT5- and KRT14-expressing basal cells are capable of extensive self-renewal and differentiation into other cells. The observed upregulation of *KRT5*, *KRT13*, and *KRT14* indicated that the injury of epithelial cells activates the proliferation and differentiation of basal cells. Additionally, we found several genes (*MMP9*, *MMP13*, *S100A8*, *S100A9*, *CSF3*, *FOS*, and *CXCL8*) involved in the IL-17 signaling pathway significantly upregulated after ammonia exposure. The IL-17 signaling pathway has been shown to play critical roles in both acute and chronic inflammatory responses. From these results, it can be concluded that inhalation of ammonia injures the nasal mucosal epithelium. The injury activated the IL-17 signaling pathway, which promotes the differentiation of basal cells to repair the epithelium.

Previous studies have shown that cilia function in concert with the airway mucus plays important roles in the innate immunity of the respiratory tract through mucociliary clearance, and defect of ciliated cells may result in mucociliary dysfunction and airway diseases [28]. In this study, we could not confirm the depletion of ciliated cells from H&E staining results due to the easy dissolution of the cilia during sample collection. However, it was interesting to find that 80 genes involved in cilium biogenesis/movement were significantly downregulated (*q* < 0.05) after ammonia exposure. Cilia are highly conserved microtubule-based organelles, and microtubules are responsible for the planar cell polarity of airway cilia and play an important role in the movement of cilia [29]. The microtubule-associated axonemal dynein motors [30] and the nexin-dynein regulatory complex [21, 31], as well as cilia- and flagella-associated protein families, are regarded as cilium-specific and essential for the assembly and stability of the ciliary architecture [32–38]. In this study, the expression levels of genes encoding axonemal dyneins (*DNAAF1*, *DNALI1*, *DNAI1*, *DNAI2*, *DNAH1*, *DNAH2*, *DNAH10*, *DNAH11*, and *DNAJB13*) and cilia- and flagella-associated proteins (*CFAP43*, *CFAP44*, *CFAP70*, and *CFAP221*) [36–38], as well as the genes involved in the assembly of the nexin-dynein regulatory complex (*DRC1*, *DRC2/CCDC65*, *DRC7*, *MAP1B*, *CCDC113*, *CCDC146*, *CCDC33*, *CCDC39*, *CCDC13*, *CCDC81*, and *CCDC96*) [21], were all significantly decreased by ammonia exposure, indicating that ammonia exposure indeed damages the structure of ciliated cells in the nasal mucosal epithelium and induces cilia dysfunction.

## Figures and Tables

**Table 1:** Downregulated genes involved in cilium biogenesis and movement.

Ensembl gene ID	Gene name	Chromosome location	Fold change (FC)^a^	padj^b^
ENSSSCG00000011078	ARMC3	10	-8.19	1.25*E*-05
ENSSSCG00000011069	ARMC4	10	-6.09	1.36*E*-04
ENSSSCG00000002356	BBOF1	7	-6.67	0.001
ENSSSCG00000015795	CCDC110	15	-4.01	7.06*E*-05
ENSSSCG00000002803	CCDC113	6	-4.36	0.011
ENSSSCG00000011291	CCDC13	13	-9.20	1.05*E*-05
ENSSSCG00000015412	CCDC146	9	-4.45	0.002
ENSSSCG00000013626	CCDC151	2	-7.35	0.001
ENSSSCG00000015109	CCDC153	9	-7.35	0.001
ENSSSCG00000026564	CCDC169	11	-2.03	0.032
ENSSSCG00000015935	CCDC173	15	-4.38	2.61*E*-06
ENSSSCG00000001908	CCDC33	7	-4.51	0.003
ENSSSCG00000011768	CCDC39	13	-2.24	0.001
ENSSSCG00000009842	CCDC60	14	-7.13	0.017
ENSSSCG00000000179	CCDC65	5	-2.83	0.002
ENSSSCG00000014917	CCDC81	9	-2.20	0.033
ENSSSCG00000028085	CCDC96	8	-5.12	0.002
ENSSSCG00000026849	CCNO	16	-3.11	0.002
ENSSSCG00000014979	CEP126	9	-2.87	3.44*E*-05
ENSSSCG00000023957	CFAP126	4	-3.31	0.009
ENSSSCG00000001786	CFAP161	7	-6.06	0.001
ENSSSCG00000015741	CFAP221	15	-6.84	0.007
ENSSSCG00000010609	CFAP43	14	-5.89	0.003
ENSSSCG00000021271	CFAP44	13	-6.39	2.18*E*-06
ENSSSCG00000017994	CFAP52	12	-5.71	0.003
ENSSSCG00000010298	CFAP70	14	-4.24	5.47*E*-04
ENSSSCG00000030513	CFAP74	6	-3.88	0.018
ENSSSCG00000005727	CFAP77	1	-7.81	0.003
ENSSSCG00000016255	DAW1	15	-9.02	3.72*E*-05
ENSSSCG00000001085	DCDC2	7	-11.22	1.54*E*-05
ENSSSCG00000014943	DEUP1	9	-4.15	1.13*E*-08
ENSSSCG00000002675	DNAAF1	6	-7.19	5.65*E*-05
ENSSSCG00000028184	DNAH1	13	-6.99	0.037
ENSSSCG00000009765	DNAH10	14	-5.30	1.31*E*-05
ENSSSCG00000015379	DNAH11	9	-3.76	2.80*E*-05
ENSSSCG00000017963	DNAH2	12	-4.21	0.009
ENSSSCG00000024185	DNAI1	10	-6.70	0.002
ENSSSCG00000024357	DNAI2	12	-9.65	0.022
ENSSSCG00000014832	DNAJB13	9	-5.93	0.001
ENSSSCG00000022979	DNALI1	6	-7.17	2.61*E*-04
ENSSSCG00000008568	DRC1	3	-5.50	6.06*E*-04
ENSSSCG00000002817	DRC7	6	-5.28	0.003
ENSSSCG00000011587	EFCAB12	13	-6.10	4.47*E*-05
ENSSSCG00000017794	EFCAB5	12	-2.61	0.048
ENSSSCG00000011205	EFHB	13	-11.99	0.016
ENSSSCG00000002620	EFHC1	7	-4.30	1.11*E*-04
ENSSSCG00000017187	FOXJ1	12	-7.91	0.01
ENSSSCG00000009877	IQCD	14	-3.00	0.031
ENSSSCG00000011855	IQCG	13	-4.30	1.10*E*-04
ENSSSCG00000016608	IQUB	18	-3.38	0.005
ENSSSCG00000015138	JHY	9	-6.51	0.002
ENSSSCG00000021571	KIF27	10	-3.46	6.42*E*-04
ENSSSCG00000001594	KIF6	7	-7.91	0.033
ENSSSCG00000004469	LCA5	1	-3.39	0.005
ENSSSCG00000028122	LCA5L	13	-3.41	0.031
ENSSSCG00000016542	LRGUK	18	-5.18	0.002
ENSSSCG00000005952	LRRC6	4	-6.05	3.39*E*-05
ENSSSCG00000001042	MAK	7	-3.50	0.002
ENSSSCG00000016974	MAP1B	16	-2.16	0.008
ENSSSCG00000011217	NEK10	13	-5.43	0.0001
ENSSSCG00000027997	NME5	2	-4.23	0.011
ENSSSCG00000006255	RP1	4	-7.48	0.0005
ENSSSCG00000006726	SPAG17	4	-8.63	3.11*E*-05
ENSSSCG00000011080	SPAG6	10	-6.00	0.0006
ENSSSCG00000025842	SPAG8	1	-2.88	0.013
ENSSSCG00000016831	SPEF2	16	-2.88	0.066
ENSSSCG00000001067	STMND1	7	-10.29	0.001
ENSSSCG00000017884	TEKT1	12	-4.22	0.007
ENSSSCG00000003633	TEKT2	6	-8.94	2.31*E*-06
ENSSSCG00000018028	TEKT3	12	-12.25	7.81*E*-06
ENSSSCG00000022389	TPPP	16	-5.98	0.001
ENSSSCG00000017415	TTC25	12	-2.76	0.004
ENSSSCG00000011263	TTC21A	13	-3.57	0.002
ENSSSCG00000000982	TTLL8	5	-3.08	0.032
ENSSSCG00000007236	TTLL9	17	-9.69	7.28*E*-04
ENSSSCG00000016216	TUBA4A	15	-6.17	3.17*E*-05
ENSSSCG00000006950	WDR63	4	-2.72	0.021
ENSSSCG00000009802	WDR66	14	-5.93	0.002
ENSSSCG00000022305	WDR78	6	-5.42	1.52*E*-04
ENSSSCG00000011408	ZMYND10	13	-6.57	3.24*E*-06

^a^Difference in gene expression between samples; -: indicates a downregulated expression. ^b^The *p* value is corrected by multiple calibration.

## Data Availability

The RNA-seq data used to support the finding of this study have been deposited in the National Center for Biotechnology Information (NCBI) SRA Database, and the accession numbers are SRX2467558, SRX2467562, SRX2467554, SRX2467548, SRX2467561, and SRX2467552.

## Supplementary Materials

Supplementary Materials Supplementary 1. Table S1: primers used for qPCR amplification. Supplementary 2. Table S2: summary of RNA-sequencing data and the number of genes per FPKM interval. Supplementary 3. Figure S1: volcano plots of differentially expressed genes. Each red point indicates an upregulated gene (*n* = 176), and each purple point indicates a downregulated gene (*n* = 426); the cutoff for the log2 fold change is 1.585.
