# Supplementary material for: Ammonia Exposure Induced Cilia Dysfunction of Nasal Mucosa in the Piglets
Source: Biomed Res Int. 2020 May 25;2020:1705387. doi: 10.1155/2020/1705387 (PMC7273420; doi:10.1155/2020/1705387)
Supplement: Supplementary Materials — Supplementary 1. Table S1: primers used for qPCR amplification. Supplementary 2. Table S2: summary of RNA-sequencing data and the number of genes per FPKM interval. Supplementary 3. Figure S1: volcano plots of differentially expressed genes. Each red point indicates an upregulated gene (n = 176), and each purple point indicates a downregulated gene (n = 426); the cutoff for the log2 fold change is 1.585. [file 1705387.f1.docx]

**Supplementary materials:**

**Supplementary 1.**

**Table S1. Primers used for qPCR amplification****.**

| Genes | Accession number | Primer sequence (5’-3’) ﹩ | Tm (°C) | Product size (bp) |
| --- | --- | --- | --- | --- |
| GAPDH | GAPDH-S | TGACATCAAGAAGGTGGTGAAG | 60 | 159 |
| (NM_001206359.1) | GAPDH-A | TTGACGAAGTGGTCGTTGAG |  |  |
| MUC5AC | MUC5AC-S | ACCAGCACCAGGTCCTCCAG | 60 | 181 |
| (XM_021082583.1) | MUC5AC-A | CAGTGCAGGGTCACGTTTCTCA |  |  |
| MUC5B | MUC5B-S | AACGAGCCGTGCTGCCT | 61 | 140 |
| (XM_021082487.1) | MUC5B-A | GGTTCGCAGCGGAATGTG |  |  |
| MMP9 | MMP9-S | AGACTCTTCACTCGGGACGGTA | 60 | 231 |
| (NM_001038004.1) | MMP9-A | GAAGGGGAAGACGCACAGGT |  |  |
| MMP12 | MMP12-S | TATGGGCTGACGATAGAAACAAC | 60 | 198 |
| (NM_001099938.1) | MMP12-A | CCTCCCTGGCATGAACATGAAAT |  |  |
| KRT14 | KRT14-S | AAGGATGCCGAGGACTGGTT | 60 | 161 |
| (XM_003482984.3) | KRT14-A | GCTTTCATGCTGAGCTGGGAC |  |  |
| FOXJ | FOXJ1-S | CACCAAGATCACCCTGTCGG | 60 | 107 |
| (XM_003357959.4) | FOXJ1-A | GAGACAGGTTGTGGCGGAT |  |  |
| DNAH10 | DNAH10-S | TGACCAAAGTCGAGGGGTTG | 60 | 169 |
| (NC_010456.5) | DNAH10-A | TGGAACAGAGGGACGTTTCC |  |  |
| TTC21A | TTC21A-S | CCACCAGTACACCAAGGCAA | 60 | 142 |
| (XM_013981474.2) | TTC21A-A | CCAGTGCCTGCTTCAAAACT |  |  |
| MAP1B | MAP1B-S | CCAATCCGGCTCTTTCTCGT | 60 | 143 |
| (XM_005672533.3) | MAP1B-A | TGGAGTTCTTCCAGTCCCCT |  |  |

﹩: The amplification efficiency of each primer has been proved to be 92%-108% by constructing standard curves.

**Supplementary 2.**

**Table S2. Summary of RNA sequencing data and the number of genes per FPKM interval.**

| Sample | Clean reads | Clean data | N (%) | Q20 (%) | Q30 (%) | GC (%) | FPKM interval | | | | |
| --- | --- | --- | --- | --- | --- | --- | --- | --- | --- | --- | --- |
|  |  |  |  |  |  |  | 0～1 | 1～3 | 3～15 | 15～60 | >60 |
| NC-1 | 120483028 | 15.06G | 0.01 | 96.78 | 92.76 | 55.59 | 10919 | 2475 | 5988 | 4209 | 1731 |
| NC-2 | 119068044 | 14.88G | 0.01 | 96.67 | 92.55 | 51.01 | 10651 | 2209 | 6121 | 4674 | 1667 |
| NC-3 | 102055914 | 12.76G | 0.01 | 96.25 | 91.69 | 53.01 | 10809 | 2360 | 6000 | 4399 | 1754 |
| D12-1 | 115188934 | 14.40G | 0.01 | 96.7 | 92.57 | 52.88 | 10815 | 2350 | 6002 | 4396 | 1759 |
| D12-2 | 105990410 | 13.25G | 0.01 | 95.88 | 92.88 | 53.88 | 10962 | 2602 | 5848 | 4095 | 1815 |
| D12-3 | 96498284 | 12.06G | 0.01 | 96.84 | 92.82 | 53.48 | 10812 | 2454 | 6072 | 4268 | 1716 |

Note: N% the percentage of the fuzzy base, Q20 base recognition accuracy rate of over 99% of the base percentage, and Q30 base recognition accuracy rate of over 99.9% of the base percentage.

**Supplementary 3.**


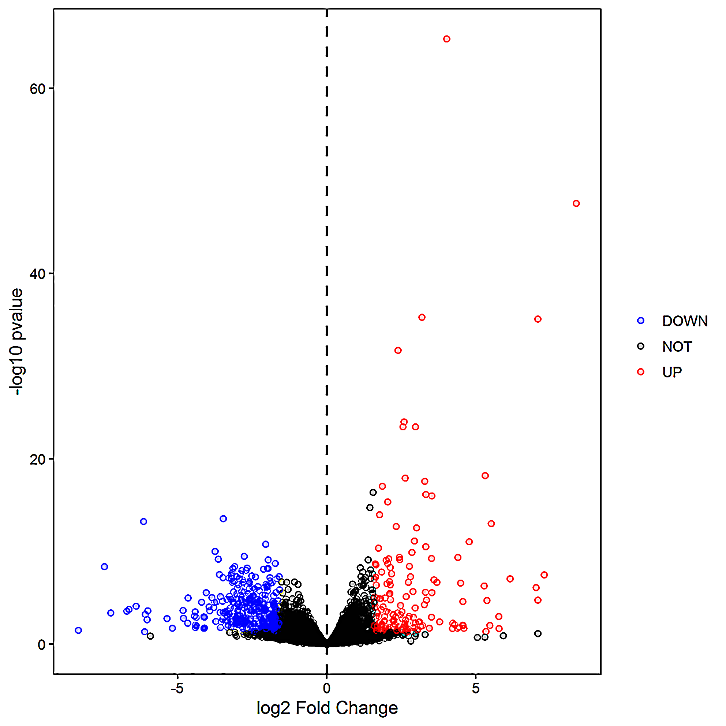


**Fig. S1 Volcano plots of differentially expressed genes.** Each red point indicates an up-regulated gene (n = 176) and each purple point indicates a down-regulated gene (n = 426); the cutoff for log2 Fold Change is 1.585.
